# SerpinE1 drives a cell-autonomous pathogenic signaling in Hutchinson–Gilford progeria syndrome

**DOI:** 10.1038/s41419-022-05168-y

**Published:** 2022-08-26

**Authors:** Giorgia Catarinella, Chiara Nicoletti, Andrea Bracaglia, Paola Procopio, Illari Salvatori, Marilena Taggi, Cristiana Valle, Alberto Ferri, Rita Canipari, Pier Lorenzo Puri, Lucia Latella

**Affiliations:** 1 IRCCS Fondazione Santa Lucia, Rome, Italy; 2 Development, Aging and Regeneration Program, Sanford Burnham Prebys Medical Discovery Institute, La Jolla, CA 92037 USA; 3 PhD Program in Cellular and Molecular Biology, Department of Biology, University of Rome “Tor Vergata”, Rome, Italy; 4 Department of Experimental Medicine, University of Rome “La Sapienza”, 00161 Rome, Italy; 5 DAHFMO, Unit of Histology and Medical Embryology, Sapienza, University of Rome, Rome, Italy; 6 Institute of Translational Pharmacology, National Research Council of Italy, Rome, Italy; 7 Present Address: BPC, Pharmakologisches Institut, Philipps-Universität Marburg, Marburg, Germany

**Keywords:** Mechanisms of disease, Senescence, Target identification, DNA damage response, Atherosclerosis

## Abstract

Hutchinson–Gilford progeria syndrome (HGPS) is a rare, fatal disease caused by *Lamin A* mutation, leading to altered nuclear architecture, loss of peripheral heterochromatin and deregulated gene expression. HGPS patients eventually die by coronary artery disease and cardiovascular alterations. Yet, how deregulated transcriptional networks at the cellular level impact on the systemic disease phenotype is currently unclear. A genome-wide analysis of gene expression in cultures of primary HGPS fibroblasts identified SerpinE1, also known as Plasminogen Activator Inhibitor (PAI-1), as central gene that propels a cell-autonomous pathogenic signaling from the altered nuclear lamina. Indeed, siRNA-mediated downregulation and pharmacological inhibition of SerpinE1 by TM5441 could revert key pathological features of HGPS in patient-derived fibroblasts, including re-activation of cell cycle progression, reduced DNA damage signaling, decreased expression of pro-fibrotic genes and recovery of mitochondrial defects. These effects were accompanied by the correction of nuclear abnormalities. These data point to SerpinE1 as a novel potential effector and target for therapeutic interventions in HGPS pathogenesis.

## Introduction

Hutchinson–Gilford progeria syndrome (HGPS) is a lethal disease caused by the autosomal dominant *de novo* mutation (1824C > T, p.G608G) in exon 11 of the *Lamin A* (*LMNA*) gene that activates a splicing donor site resulting in the production of Progerin, a toxic LMNA variant [1, 2] that leads to premature aging features [3, 4]. HGPS patients share essential molecular and clinical features with physiological aging, making HGPS a condition that prematurely recapitulates the aging process [5, 6]. At the clinical level, HGPS individuals display atherosclerosis [7], osteoporosis, loss of subcutaneous fat, reduced bone density and cardiovascular alterations [8] resulting in myocardial infarction and stroke, the main causes of their premature death within the second decade of life [9].

At cellular level, HGPS patients exhibit abnormal nuclear shape [10], loss of peripheral heterochromatin [11, 12], DNA damage accumulation [13, 14], telomere shortening [14, 15], genomic instability [16] and acquisition of senescence phenotype [17] leading to altered patterns of gene expression [18]. The nuclear defects interfere with cellular functions including the inflammatory response, metabolic changes, proteostasis and mitochondrial dysfunction [19, 20]. Altogether, the cellular defects are responsible for the pathological features that mark progeroid syndrome. However, how deregulated transcriptional networks at the cellular level impact on the systemic disease phenotype remains unclear.

Targeting mechanosensing processes mediated by lamins has been shown to be an efficient strategy to protect cells from nuclear ruptures, DNA damage and cell cycle arrest [21]. Indeed, lamins and the linker of the nucleoskeleton and cytoskeleton (LINC) complex transmit forces from the extracellular matrix (ECM) into the nucleus [22]. Conceivably, the integrity of nuclear lamina is required for reciprocal transmission of nuclear forces back to the ECM, as indicated by the widespread alterations of cellular and tissue functions observed in HGPS patients [23]. Indeed, Progerin expression in vascular smooth muscle and endothelial cells leads to arterial stiffness and cardiovascular pathology in HGPS [24, 25]. Therefore, altered signaling from the nucleus to the ECM could be a key event in HGPS pathogenesis. However, the cellular effectors of pathological nuclear-to-ECM signaling in HGPS cells are unknown to date and this prevents the development of effective strategies to counter HGPS progression and deleterious outcomes.

Here, we report on the identification of SerpinE1 (also known as PAI-1) as a molecular effector that propels a cell-intrinsic pathogenic signaling in HGPS patient-derived primary fibroblasts. SerpinE1 functions as the primary inhibitor of plasminogen activators (PA) - tissue plasminogen activator (tPA) and urokinase (uPA) – which, in cooperation with matrix metalloproteinases (MMPs), affects fibrinolysis and other ECM properties [26, 27]. SerpinE1 can be transcribed through several signaling cascades, including pro-fibrogenic and pro-inflammatory pathways [26]. Accordingly, increased levels of SerpinE1 have been reported in metabolic diseases [28] and are typically connected to age-associated increase in occurrence of thrombosis [29] and development of atherosclerosis [30]. Conversely, lower SerpinE1 levels have been linked to protection against physiological aging [31, 32] and SerpinE1 deficiency overcome senescence [33]. Hence, SerpinE1 can be considered as a putative marker and mediator of cellular senescence [34], as also indicated by its presence among the main “core components” of the senescence-associated secretory phenotype (SASP) typicaly exhibited by senescent cells [35]. More recently, systemic SerpinE1 concentration has been proposed as a biomarker of age-related processes in non-human primates [36]. Furthermore, the SerpinE1 inhibitor TM5441 protects against development of pathogenic features of cellular senescence [37], is effective against high-fat diet-induced obesity and adipocyte injury [38] and attenuates hypertension, cardiac hypertrophy, and periaortic fibrosis [39, 40].

Here, we provide evidence that SerpinE1 is a cell-autonomous molecular player of pathogenic features associated to HGPS development and progression. Therefore, targeting SerpinE1 might provide a valid approach to prevent and/or delay cellular morbidity hallmarks in HGPS.

## Results

### Transcriptional signature in HGPS primary fibroblasts

To identify altered patterns of gene expression in HGPS, we performed RNA-sequencing (RNA-seq) analysis in primary HGPS dermal fibroblasts isolated from 2-year- and 8-year-old HGPS patients (2YO HGPS and 8YO HGPS, respectively) analyzed at passage 15. As a control, we used primary human fibroblasts from aged-matched healthy donors (2YO Ctrl and 8YO Ctrl).

HGPS fibroblasts showed flat morphology and exhibited increased beta-Galactosidase activity (beta-Gal) (Fig. S1A), two typical features of cellular senescence. Consistently with previous studies showing reduced proliferative capacity and premature acquisition of senescence features in progerin-expressing cells, in HGPS primary fibroblasts we measured increased levels of *p16/INK4* and *p21/Waf1* at both transcriptional (Fig. S1B) and protein level (Fig. S1C, D). Between passage 13 and 15, the cellular stages in which we performed all of our analyses, both 2YO and 8YO HGPS fibroblasts displayed nuclear abnormalities and precocious activation of DNA damage signaling, evaluated by immunofluorescence for H2AX phosphorylated in Serine 139 (γ-H2AX), NbsI phosphorylated in Serine 343 (P-NbsI) (Fig. S1E, F) and 53BP1 (Fig. S1G). Activation of DNA damage response was also measured by Western blot for the downstream DNA damage effector, P53 phosphorylated in Serine 15 (Ser15 P53) (Fig. S1C), further confirming that our in vitro system recapitulates the progerioid pathology at cellular level.

**Fig. 1:**
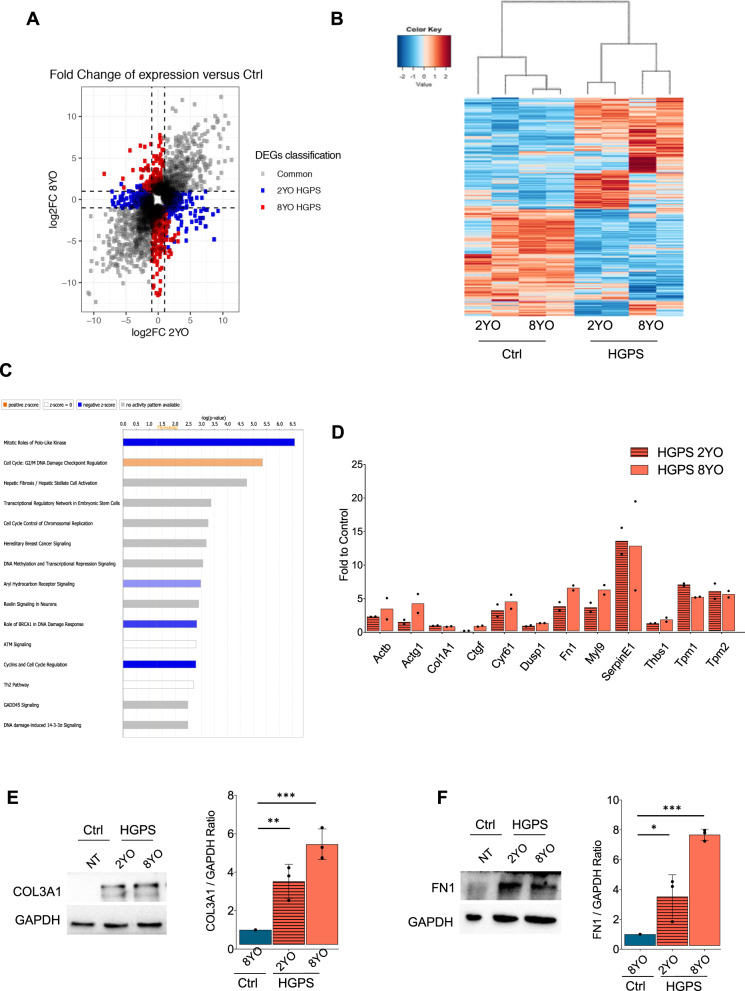
Transcriptional signature in HGPS primary fibroblasts. **A** Scatterplot of differentially expressed genes (DEGs) of 2YO and 8YO HGPS fibroblasts. Common genes of 2YO and 8YO HGPS are shown in gray, 2YO HGPS unique genes in blue and 8YO HGPS unique genes in red. **B** Heatmap of DEGs from 2YO and 8YO HGPS vs. age-matched control cells (2YO Ctrl and 8YO Ctrl). **C** Ingenuity Pathway Analysis (IPA) showing enriched categories of common DEGs in 2YO and 8YO HGPS fibroblasts. **D** qRT-PCR of genes associated with fibrosis: *Actb* (actin beta), *Actg1* (actin gamma 1), *Col1A1* (Collagen Type I Alpha 1 Chain), *Ctgf* (Connective tissue growth factor), *Dusp1* (Dual Specificity Phosphatase 1), *Fn1* (Fibronectin1), *Myl9* (Myosin Light Chain9), *SerpinE1*, *Thbs1* (Thrombospondin 1), *Tpm1* (Tropomyosin 1), *Tpm2* (Tropomyosin 2) in 2YO and 8YO HGPS cells compared to 8YO control cells. **E** Cropped immunoblot for Collagen3A1 (COL3A1) and GAPDH in 8YO control (Ctrl) and 2YO and 8YO HGPS. Plot represents COL3A1/GAPDH ratio based on the average for each experimental point, (*n* = 3). **F** Cropped immunoblot for Fibronectin1 (FN1) and GAPDH in 8YO control (Ctrl) and 2YO and 8YO HGPS. Plot represents FN1/GAPDH ratio based on the average for each experimental point, (*n* = 3).

RNA-seq analysis identified 1744 and 2059 differentially expressed genes (DEGs) from the 2YO HGPS vs. 2YO Ctrl, and 8YO HGPS vs. 8YO Ctrl comparisons, respectively. The majority of DEGs were shared between 2YO and 8YO HGPS samples, with 783 common genes between the two conditions (Fig. 1A). Heatmap showed that control samples (2YO Ctrl and 8YO Ctrl) clustered separately from HGPS samples (2YO HGPS and 8YO HGPS) (Fig. 1B). Gene ontology (GO) for the common DEGs revealed that the enriched categories were related to fibrosis, DNA damage, checkpoint activation and deregulation of the cell cycle, according to the progeroid phenotype displayed by HGPS cells (Fig. 1C).

Given that fibrosis was one of the most enriched categories extrapolated from the GO analysis, the expression of a subset of pro-fibrotic genes found upregulated in our RNA-seq analysis was validated by qRT-PCR in 2YO and 8YO HGPS fibroblasts. We detected increased levels of all analyzed genes in HGPS fibroblasts, as compared to age-matched control cells (Fig. 1D). Consistently, we measured the accumulation of Collagen3A1 and Fibronectin1, indicating increased collagen deposition (Fig. 1E, F). In particular, our attention was captured by SerpinE1 (also known as PAI-1) whose expression was detected to increase up to 20-fold in HGPS fibroblasts (Fig. 1D).

### SerpinE1 activity increases in HGPS primary cells

Analysis of publicly available datasets generated in HGPS fibroblasts revealed a loss of LMNA binding to SerpinE1 promoter [41], suggesting that SerpinE1 expression is induced as a direct consequence of loss of methylation on its promoter due to alteration in the nuclear lamina, rather than secondary events developed in cultured HGPS fibroblasts.

To assess the aberrant activation of SerpinE1 in progeria samples, we first evaluated its expression at the transcriptional level. SerpinE1 expression increased in all HGPS primary cells that we tested compared to control cells (Fig. S2A). We analyzed HGPS cells derived from patients at different ages, ranging from 2 to 8 years. Functional activation of SerpinE1 was evaluated by reverse-zymography (Fig. S2B–E). In this assay, the presence of the inhibitory effect of unbound SerpinE1 was detected by the increased intensity of bands representing areas resistant to lysis (Fig. S2B). These data are in agreement with the high SerpinE1 levels found in HGPS cells (Fig. 1D, Fig. S2A). Next, we evaluated the presence of Plasminogen Activator (PA) proteolytic activity by both zymography (Fig. S2C) and by chromogenic substrate assay (Fig. S2D). Furthermore, the “lytic areas” were plasminogen-dependent and were inhibited by amiloride, a specific inhibitor of uPA (Fig. S2E). Collectively, these data show a decreased uPA activity in HGPS cells, as a consequence of increased SerpinE1 expression.

### SerpinE1 downregulation reverts HGPS pathological features

**Fig. 2:**
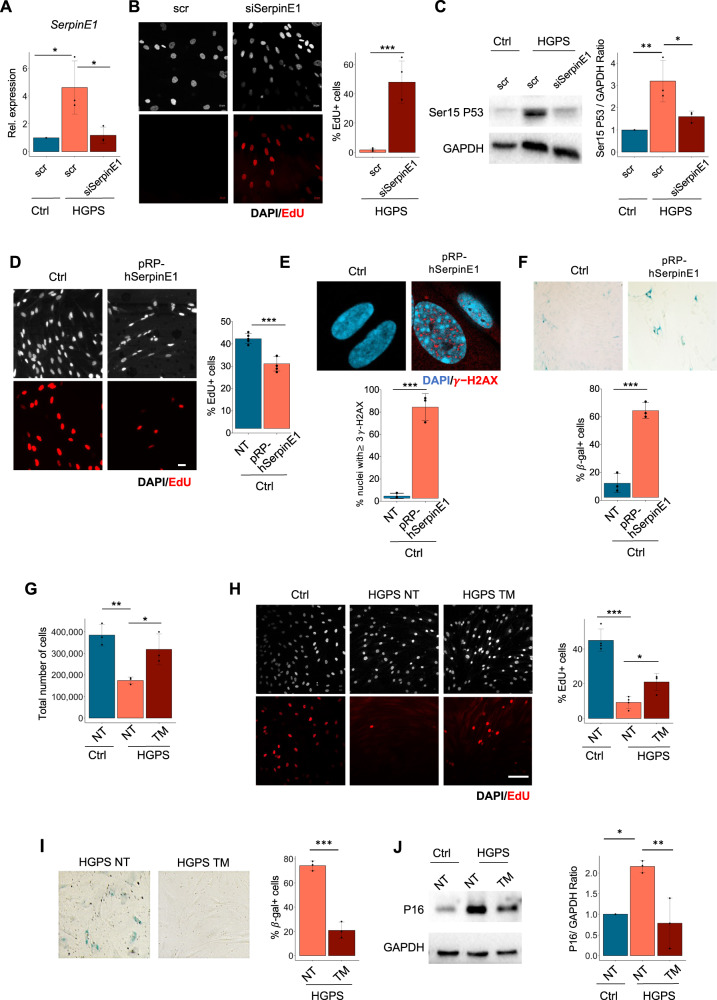
SerpinE1/PAI-1 downregulation resumes proliferation and reduces DNA damage in HGPS. **A** qRT-PCR for SerpinE1 transcript after 48 h upon transfection in control cells (Ctrl) and HGPS transfected with scramble (scr) or siRNA for SerpinE1 (siSerpinE1), (*n* = 3). **B** Representative images relative to 8YO HGPS fibroblasts transfected with scramble (scr) or siRNA for SerpinE1 (siSerpinE1) analyzed 5 days post transfection and exposed to a 16 h EdU pulse (red). Scale bar 20 μm. Quantification of the percentage EdU positive nuclei in 8YO HGPS fibroblasts transfected with scramble (scr) or siRNA for SerpinE1(siSerpinE1) analyzed 5 days post transfection and exposed to a 16 h EdU (*n* = 3). **C** Cropped immunoblot for p53 phosphorylated at Serine 15 (Ser15 P53) and GAPDH as loading control in 8YO HGPS fibroblasts transfected with scramble (scr) or siRNA for SerpinE1 (siSerpinE1) analyzed 5 days post transfection. Plot represents Ser15 P53/GAPDH ratio based on the average for each experimental point, (*n* = 3). **D** Representative images of healthy fibroblasts transfected four consecutive times every 3 days with a Ctrl vector pcDNA3 (Ctrl) and pRP[Exp]-CAG > hSERPINE1 (pRB-hSerpinE1) and exposed to a 6 h EdU pulse (red). Scale bar 20 μm. Quantification of the percentage of EdU positive nuclei in Ctrl and pRB-SerpinE1 fibroblasts exposed to a 6 h EdU pulse, (*n* = 4). **E** Representative images of immunofluorescence for γ-H2AX (red) and DAPI (blue) in healthy fibroblasts transfected four consecutive times every 3 days with a Ctrl vector pcDNA3 (Ctrl) and pRP[Exp]-CAG > hSERPINE1 (pRB-hSerpinE1). Scale bar 20 μM. Quantification of the percentage of nuclei in Ctrl and pRB-SerpinE1 fibroblasts with at least three γ-H2AX foci, (*n* = 3). **F** Beta-Gal staining in healthy fibroblasts transfected four consecutive times every 3 days with a Ctrl vector pcDNA3 (Ctrl) and pRP[Exp]-CAG > hSERPINE1 (pRB-hSerpinE1). Quantification of the percentage of beta-gal positive cells in Ctrl and pRB-SerpinE1 fibroblasts, (*n* = 3). **G** Plot representing the total cell number of 8YO HGPS fibroblasts untreated and treated with TM5441 (TM) for 10 days compared to the age-matched control (Ctrl). **H** Representative images relative to 8YO HGPS fibroblasts untreated and treated with TM5441 (TM) for 10 days and age-matched control (Ctrl) exposed to a 16 h EdU pulse (red). Scale bar 50 μm. Quantification of the percentage EdU positive nuclei in 8YO HGPS fibroblasts untreated and treated with TM5441 (TM) for 10 days and age-matched control (Ctrl) exposed to a 16 h EdU pulse, (*n* = 4). **I** Beta-Gal staining in 8YO HGPS fibroblasts untreated (NT) or treated with TM5441 (TM). Quantification of the percentage of beta-gal positive cells from 8YO HGPS fibroblasts untreated (HGPS NT) or treated with TM5441 (HGPS TM), (*n* = 3). **J** Cropped immunoblot for P16 and GAPDH in 8YO HGPS (HGPS) untreated and treated with TM5441(TM) and age-matched control (Ctrl) cells. Plot represents P16/GAPDH ratio based on the average for each experimental point, *n* = 3.

To investigate the function of SerpinE1 in HGPS fibroblasts, we evaluated the outcome of its downregulation by siRNA (Ambion, ID #s10015) (Fig. 2A). SerpinE1 downregulation in 8YO HGPS fibroblasts resumes the cell cycle, as revealed by 5-ethynyl2'-deoxyuridine (EdU) incorporation (Fig. 2B), and reduces DNA damage signaling, a cardinal pathological trademark of HGPS cells, evaluated by Ser15-P53 (Fig. 2C). On the other hand, we performed gain of function experiments by overexpression of SerpinE1 in healthy fibroblasts that resulted in reduction of cell proliferation, as assessed by EdU incorporation (Fig. 2D), together with increase DNA damage accumulation (Fig. 2E) and beta-Gal activity (Fig. 2F). Thus, downregulation in HGPS cells or forced overexpression of SerpinE1 in normal fibroblasts inhibits or trigger, respectively, pathological features of the HGPS.

**Fig. 3:**
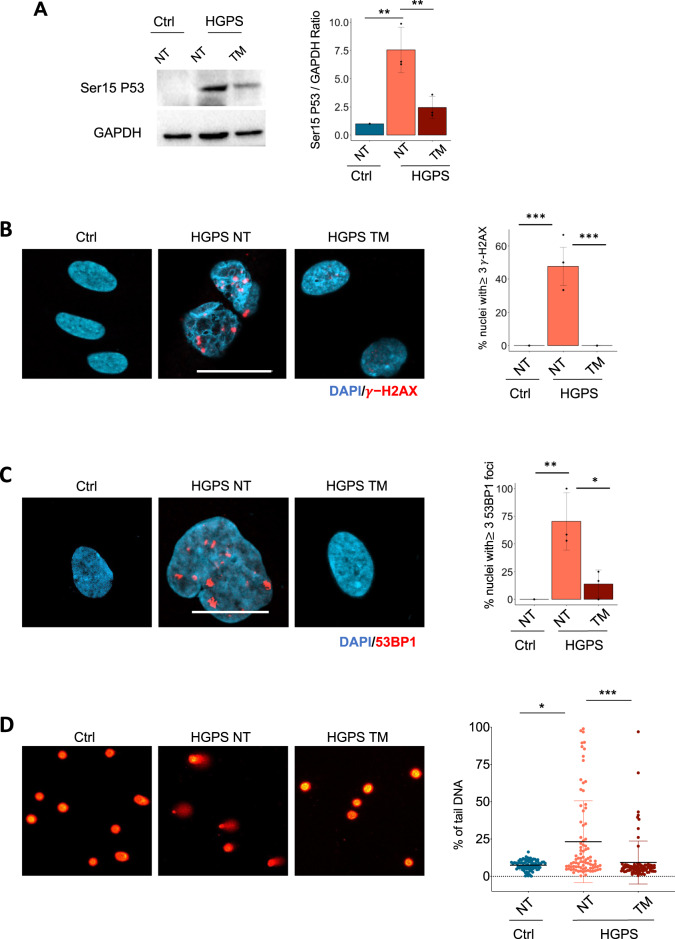
TM5441 treatment reverts pathological features associated to HGPS. **A** Cropped immunoblot for p53 phosphorylated at Serine 15 (Ser15 P53) and GAPDH as loading control in 8YO HGPS untreated and treated with TM5441(TM) and age-matched control (Ctrl) cells. Plot represents Ser15 P53/GAPDH ratio based on the average for each experimental point (*n* = 3). **B** Representative images of immunofluorescence for γ-H2AX (red) and DAPI (blue) in 8YO HGPS fibroblasts untreated (NT) or treated with TM5441 (TM) and age-matched control (Ctrl). Scale bar 30 μM. Quantification of the percentage of nuclei from 8YO HGPS fibroblasts untreated (NT) or treated with TM5441 (TM) and age-matched control (Ctrl) with at least three γ-H2AX foci, (*n* = 3). **C** Representative images of immunofluorescence for 53BP1 (red) and DAPI (blue) in 8YO HGPS fibroblasts untreated (NT) or treated with TM5441 (TM) and age-matched control (Ctrl). Scale bar 30 μM. Quantification of the percentage of nuclei from 8YO HGPS fibroblasts untreated (NT) or treated with TM5441 (TM) and age-matched control (Ctrl) with at least three 53BP1 foci, (*n* = 3). **D** Representative images of nuclei assayed for Alkaline Comet in 8YO HGPS fibroblasts untreated (NT) or treated with TM5441 (TM) and age-matched control (Ctrl). Quantification of Alkaline Comet Assay performed in control fibroblasts (Ctrl), 8YO HGPS untreated and treated with TM5441 (TM) for 10 days. The percentage of tail DNA values is represented in the graphs.

We therefore sought to test a pharmacological treatment that reduces SerpinE1 activity. Among the known small molecules shown to counter SerpinE1 activity, we selected the SerpinE1 inhibitor TM5441, an orally bioavailable compound employed at the concentration of 10 μM for 10 days as already described [39, 42]. When we applied the same protocol to HGPS fibroblasts, we measured the ability of TM5441 treatment to increase uPA activity (Fig. S3A), suggesting that TM5441 treatment might be used to reduce SerpinE1 aberrant activation in HGPS cells. We then monitored the outcome of pharmacological inhibition of SerpinE1 activity by TM5441 on several cell-intrinsic pathological features exhibited by HGPS fibroblasts such as cell cycle arrest, senescence, and DNA damage accumulation. We first observed that TM5441 treatment restores the proliferative potential of HGPS human fibroblasts, as assessed by measuring the growth rate (Fig. 2G) and EdU incorporation (Fig. 2H). Likewise, the treatment with TM5441 was efficient in mitigating senescence, as revealed by reduced amount of Beta-Gal (Fig. 2I) as well as in lowering P16 levels (Fig. 2J). Next, we measured DNA damage signaling, revealed by Ser15-P53 levels, that we found attenuated in TM5441-treated HGPS fibroblasts, as compared to age-matched control cells (Fig. 3A). Consistently, TM5441 treatment could also significantly reduce γ-H2AX (Fig. 3B) and 53BP1 (Fig. 3C) foci within HGPS cells. As γ-H2AX is a typical event stimulated by DNA damage response (DDR), we used alkaline Comet assay to monitor the DNA damage repair ability at the single-cell level. Indeed, TM5441 treatment led to a significant reduction of DNA lesions in HGPS fibroblasts at levels similar to the one of control cells (Fig. 3D).

### SerpinE1 inhibition restores normal nuclear shape and erases the fibrotic signature

**Fig. 4:**
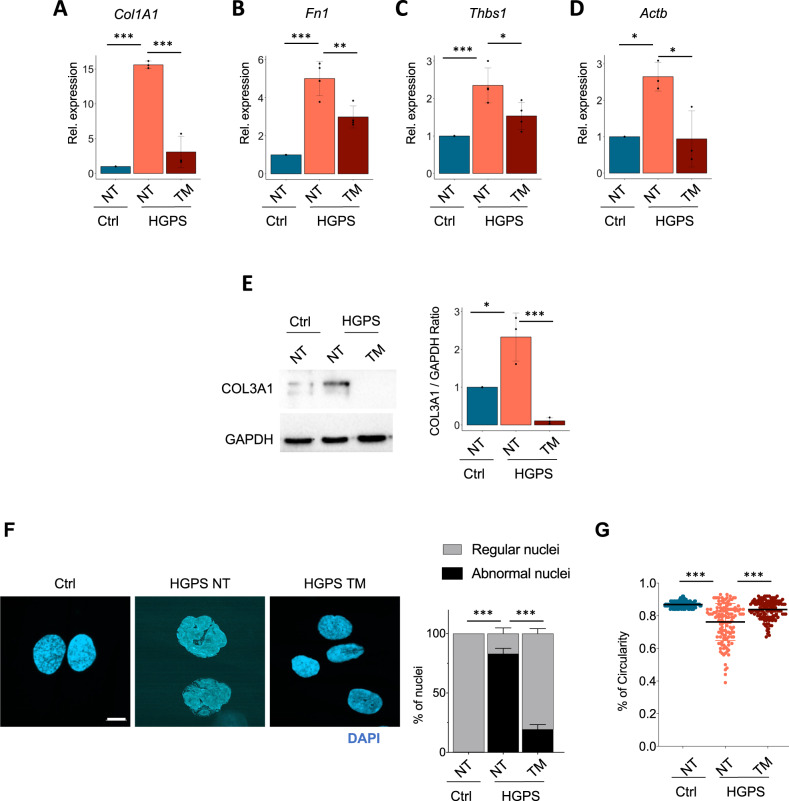
TM5441 treatment restores normal nuclear shape and reduces the accumulation of fibrosis. **A**–**D** qRT-PCR for *Col1A1* (*n* = 3), *Fn1* (*n* = 4), *Thbs1* (*n* = 4) *and Actb* (*n* = 3) in control (Ctrl) and 8YO HGPS untreated and treated with TM5441 (TM). **E** Cropped immunoblot for Collagen3A1 (COL3A1) and GAPDH in 8YO HGPS untreated and treated with TM5441(TM) and age-matched control (Ctrl) cells. Plot represents COL3A1/GAPDH ratio based on the average for each experimental point, (*n* = 3). **F** Representative images of immunofluorescence for DAPI (blue) in 8YO HGPS fibroblasts untreated (NT) or treated with TM5441 (TM) and age-matched control (Ctrl). Scale bar 30 μM. Quantification of regular and abnormal nuclei in control (Ctrl) and 8YO HGPS cells untreated (NT) or treated with TM5441 (TM), (*n* = 3). **G** Quantification of nuclear circularity in control (Ctrl) and 8Y HGPS cells untreated (NT) or treated with TM5441 (TM).

As an inhibitor of fibrinolysis, SerpinE1 is considered a pro-fibrogenic gene which is part of a larger list of pro-fibrotic genes upregulated in HGPS fibroblasts (Fig. 1). Indeed, we found that several pro-fibrotic genes, including collagen1A1 (Fig. 4A), fibronectin1 (Fig. 4B), thrombospondin 1 (Fig. 4C), actin beta (Fig. 4D) transcripts and Collagen3A1 protein (Fig. 4E), were upregulated in HGPS fibroblasts. Interesting, upon treatment with the SerpinE1 inhibitor TM5441, their expression was restored to levels typically observed in control normal fibroblasts (Fig. 4A-E), suggesting that upregulation of SerpinE1 is a key pathogenic event required for the activation of other effectors of the pro-fibrotic phenotype exhibited by HGPS fibroblasts.

In agreement with the Progerin-mediated alteration of the nuclear lamina, we measured the ability of TM5441 treatment to affect cell nuclear shape. We quantified normal (not blebbed) vs. abnormal (blebbed) nuclei showing that the majority of HGPS cells treated with SerpinE1 inhibitor displayed a normal nuclear shape (Fig. 4F). The morphological nuclear abnormalities were also estimated using the nuclear contour ratio [43], which measures how a shape deviates from a circle, with 1 representing a perfect circle and decreasing values representing an increasing level of deformation. The mean nuclear contour ratio of HGPS cell values was 0.7628. By contrast, the mean nuclear contour ratio of HGPS treated with TM5441 was significantly higher (0.8375; *P*-value < 0.0001) (Fig. 4G).

### TM5441 treatment reverts mitochondrial defects in HGPS

**Fig. 5:**
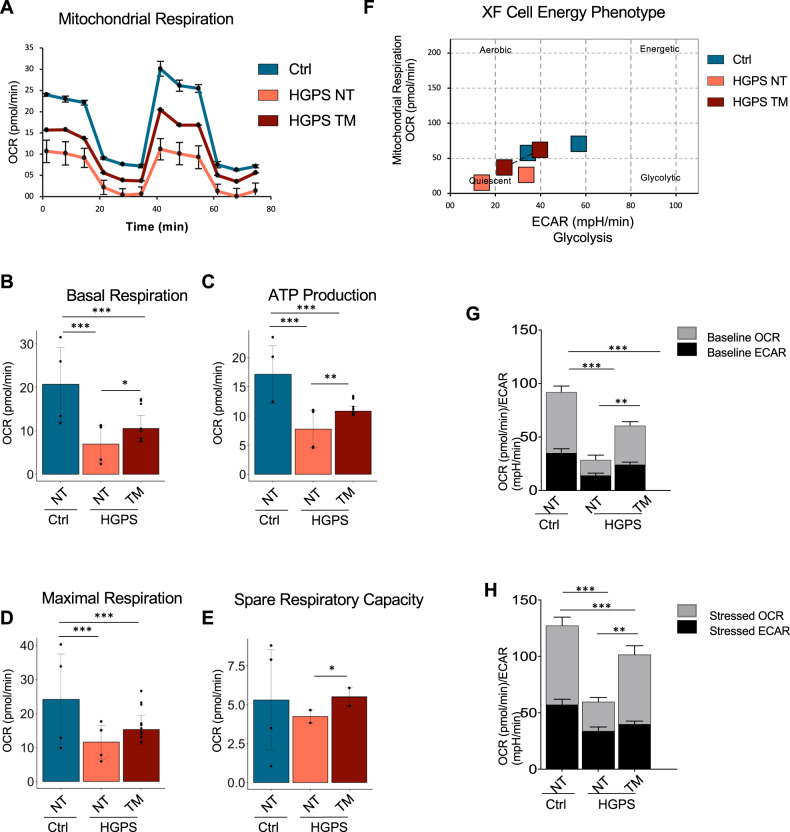
TM5441 treatment improves energy metabolism of HGPS. **A** Representative data graphic of oxygen consumption rate (OCR) obtained from the Cell Mito Stress Test. Mitochondrial respiration expressed as normalized OCR of control, HGPS and HGPS TM5441-treated cells after the sequential addition of oligomycin, FCCP, rotenone and antimycin as indicated. **B** The basal respiration which represents the oxygen consumption used to meet cellular ATP demand in resting condition. **C** Mitochondrial ATP production, calculated as the OCR difference between the baseline and after the addition of oligomycin. **D** The maximal respiration measured as the maximal oxygen consumption rate attained by adding the uncoupler FCCP. **E** The spare respiratory capacity obtained from the analysis of the Maximal respiration. **F** The cellular energy diagram showing the normalized oxygen consumption rate (OCR) and the normalized extracellular acidification rate (ECAR) of control, HGPS and HGPS TM5441-treated cells at baseline and stressed conditions. **G**, **H** Metabolic potential of control, HGPS and HGPS TM5441-treated fibroblasts was calculated from unstressed **G** and stressed (**H**) OCR/ECAR over baseline.

Mitochondrial defects contribute to premature organ decline and aging in HGPS and constitute one of the cell-autonomous disfunction associated to progeroid diseases [20]. Indeed, recovery of mitochondrial function through ROCK inhibition proved to ameliorate HGPS phenotype [44]. We therefore evaluated the ability of TM5441 in affecting mitochondrial functionality, by performing a Mito Stress Test using the Seahorse^®^XFe Technologies. This assay allows the analysis of a wide panel of mitochondrial bioenergetic parameters through the real-time measurement of oxygen consumption rate (OCR) in live cultured cells and enabled us to evaluate the differences in the energy profile between TM5441-treated and untreated HGPS cells. As described by representative OCR profiles, TM5441 treatment improved the bioenergetic performances of HGPS cells (Fig. 5A). Data analysis showed a significant difference in basal respiration between treated and untreated HGPS cells. Indeed, HPGS cells showed lower basal OCR that was partially reverted by TM441 treatment (Fig. 5B).

We inferred ATP production by analyzing the difference between the basal oxygen consumption and values measured after oligomycin injection. HGPS fibroblasts showed a significant drop in ATP production that was partially reverted by TM5441 treatment (Fig. 5C). The addition of phenylhydrazone (FCCP), dissipating the protonic gradient, maximizes the electron flow through the mitochondrial transport chain and increases oxygen consumption, which indicates the efficiency of the electron flow. The inability of HGPS cells to maximize the OCR following FCCP was partially abrogated by the TM441 treatment (Fig. 5D). Similarly, the spare respiratory capacity (SRC), a measure of the ability of the cell to respond to increased energy demand was improved in HGPS cells upon TM441 treatment (Fig. 5E). Indeed, FCCP mimics a physiological “energy demand” by stimulating the respiratory chain to operate at maximum capacity, which causes rapid oxidation of substrates to meet metabolic challenges.

These results are summarized by the Cell energy phenotype map (Fig. 5F) that clearly indicates a net improvement in the energy metabolism of HGPS cells treated with TM441 as further shown by the analysis of the OCR/ECAR (extracellular acidification rate) ratio performed with or without stressors (Fig. 5G, H).

## Discussion

To date, there is no cure for HGPS patients, although in 2020 farnesyltransferase inhibitor (FTI) lonafarib became the first (and only) FDA-approved drug to treat progeria [45]. This scenario sets the urgency to search for additional candidate drugs or strategies aimed at restoring cellular homeostasis in HGPS. Many therapeutic approaches are developing to treat patients with HGPS [46], including gene therapies, which exploit CRISPR/Cas9 [47, 48], in vivo adenine base editors (ABEs) [49] and small molecules that display different biological features. Several reports highlight the ability of rapamycin, or its analogs everolimus, to enhance cell proliferation, reduce nuclear blebbing and progerin aggregates and delay the onset of cellular senescence [50]. The beneficial effects of genetic mTOR reduction in double-copy LMNA G608G transgenic mice provide additional indication that mTOR inhibition might counter progeroid disease [51]. As such, the identification of pharmacological approaches with available compounds that could be immediately translated to patients is an urgent and imperative task to counter disease progression, improve quality of life and extend lifespan of HGPS patients [46].

Increased evidences highlight the central role of nuclear defects in affecting cellular functions such as inflammatory response, metabolism, proteostasis and mitochondrial activity [6, 52]. Since altered vascular homeostasis appears to be the major cause of cardiovascular alterations in HGPS [53], most research has focused on dysfunctional activities of endothelial or other vessel-associated cells types. Still, nuclear defects are present in all cell types of HGPS individuals, thereby prompting an interest on the potential contribution of other cell types to HGPS-related cardiovascular pathogenesis. Among these cells, fibroblasts are major candidates as they regulate angiogenesis and vascular homeostasis directly, by releasing soluble vasoactive factors, or indirectly, by altering ECM composition. Consistently, the majority of cardiovascular diseases (CVD) are associated with excessive ECM deposition of pro-fibrotic components and vascular stiffening, which ultimately determine pathological cardiac fibrosis [54]. Maintenance of proper mechanotransduction in healthy cells is warranted by a complex interplay between ECM components and nuclear lamins. Presumably, alterations of this interplay can contribute to the development of cell-autonomous pathogenic events in HGPS fibroblasts. Our RNA-seq analysis in primary fibroblasts derived from HGPS patients (as compared to their healthy counterpart) has identified SerpinE1 upregulation and functional activation as a key mediator of a reciprocal signaling between nuclear lamina and aberrant activation of pathogenic events that contribute to the disease development, including constitutive DNA damage, mitochondrial defects and expression of pro-fibrotic genes. Indeed, either siRNA-mediated or pharmacological inhibition of SerpinE1 could invariably recover nuclear lamina integrity and reverse mithocondrial defects, activation of DNA damage and expression of pro-fibrotic genes, eventually restoring cellular homeostasis. Thus, SerpinE1 upregulation appears to be a rate-limiting event for the development of key pathogenic features of HGPS. As deregulated expression of SerpinE1 has been found in several age-associated metabolic diseases [28] as well as in HGPS-associated pathological events, such as thrombosis [29] and atherosclerosis [30], it is tempting pointing to SerpinE1 upregulation as a key event in HGPS development and progression.

Thus, while we acknowledge the limitation of the findings of the present work which were based only on in vitro analysis of human primary fibroblasts derived from HGPS patients, our data suggest that SerpinE1 inhibition (e.g. by TM441) could be exploited as a potential therapeutic tool to treat HGPS.

### Experimental procedures

#### Cell culture and treatments

To study DNA damage accumulation, we used human dermal fibroblasts obtained by the Progeria Research Foundation. We employed human fibroblasts isolated from 2YO (HGADFN003 and HGADFN 188) and 8YO (HGADFN167 and HGADFN169) HGPS patients, diagnosed by the detection of the c.1824C > T (p.Gly608Gly) heterozygous LMNA pathogenic variant. 8YO HGPS fibroblasts provided by Coriell Cell Repositories (AG11513) were also employed in this study. Other cell line (HGADFN127 and HGADFN367) isolated from 3YO were used to validate data. As a control we used fibroblasts derived from aged-matched healthy donors (2YO Ctrl - AG07095 - and 8YO Ctrl - GM08398 -) provided by Coriell Cell Repositories. Cells were cultured in growth medium, Dulbecco’s modified Eagles medium (DMEM) (Life Technologies) supplemented with 15% fetal bovine serum (FBS) (Hyclone) and penicillin/streptomycin at 37 °C. Fibroblasts were cultured on 20 and 56 cm^2^ plates and allowed to reach 90% confluence, then trypsinized and splitted 1:2. For all the experiments described, we harvested cells at passage 13 to 15. For siRNA transfection, HGPS cells were transfected with siRNA for SerpinE1 (Ambion, ID #s10015), using jetOPTIMUS® (Polyplus, 117-01) according to manufacturer instructions; after 4 h the medium was replaced with growth medium. For SerpinE1 overexpression experiments, healthy fibroblasts were transfected with either pcDNA3 control vector and pRP[Exp]-CAG > hSERPINE1[NM_001386460.1]:BGH pACMV > EGFP(ns):T2A:Puro (vector ID: Vector Builder 220605-1027sgw). In order to keep high level of SerpinE1 we performed four consecutive (every 3 days) rounds of transfections. To inhibit SerpinE1/PAI-1, we treated fibroblasts with TM5441 (Tocris) at concentration of 10 µM for 10 days, refreshed every other day.

#### Immunofluorescence

In order to follow DNA damage accumulation, we performed immunofluorescence assays in HGPS and age-matched control cells plated on coverslips and fixed with 4% paraformaldehyde or methanol/acetone for 10 min at room temperature. Fixed cells were permeabilized with 0.25% TritonX-100 in PBS and nonspecific antibody-binding sites were blocked by incubation with 4% BSA in PBS for 1 h. The following primary antibodies were used: γ-H2AX (Millipore, 05-636), P-Nbs1 (Epitomics, 2194), 53BP1 (Novus Biologicals, NB100-304). Immunostaining with primary antibodies was performed overnight at 4 °C. Antibody binding was revealed using species-specific secondary antibodies coupled to Alexa Fluor 488 or 594 (Molecular Probes, Eugene, CA, USA). DNA synthesis was evaluated by EdU incorporation following the manufacturer’s instructions (Invitrogen). Nuclei were visualized by DAPI (4′,6′-diamino-2-phenylindole). Images were acquired using Zeiss LSM5 Pascal confocal microscope. Fields reported in the figures are representative of all examined fields. Images were assembled using ImageJ software (NIH, Bethesda, MD, USA) and Photoshop software (Adobe Systems Incorporated, San Jose, CA, USA). Immunofluorescence images are representative of at least three different experiments.

#### Protein extraction and Western blot

Proteins were extracted with RIPA buffer supplemented with PMSF and protease inhibitor cocktail as described in ref. [55] and resolved in SDS polyacrilamide gels, then transferred to nitrocellulose membranes (Bio-Rad). Western blot was performed using antibodies against the following proteins: P16 (Santa Cruz Biotechnology, C-20), P21 (Santa Cruz Biotechnology, C-19), Ser15 P53 (Cell Signaling, 9284), Collagen3A1 (Santa Cruz, sc-271249), Fibronectin1 (Genetex, GTX112794). GAPDH (Cell Signaling, D16H11 5174 S) and tubulin (Neo Markers, Ab4) were used as protein normalization markers. HRP-conjugated secondary antibodies were revealed with the ECL chemiluminescent kit (Amersham) following the manufacturer’s instructions. The signal was detected with a ChemiDoc MP Imaging System (Bio-Rad). Western blot analysis are representative of at least three different experiments.

#### RNA extraction and RT–PCR

Total RNA was extracted using TRI Reagent (Sigma), according to the manufacturer’s instructions. RNA (0.5–1 µg) was retrotranscribed using the TaqMan reverse transcription kit (Applied Bio-systems). qRT-PCR was performed to analyze relative gene expression levels using SYBR Green master mix (Applied Biosystems). Relative gene expression was normalized for TBP expression value and calculated using the 2^ΔΔCT^ method. For progerin amplification we performed a semiquantitative PCR following these conditions: 1 cycle of 2 min at 50 °C; 1 cycle of 10 min at 95 °C; and 40 cycles of 15 s at 95 °C, 15 s at 58 °C, and 30 s at 72 °C. The relative expression values for progerin were normalized to GAPDH. Primers sequences are listed in Table 1. Real-Time PCR analysis are representative of at least three different experiments.

**Table 1:** Primers for qPCR.

Table 1	Primer list	
Gene	FWD	REV
*ACTB*	CGTCTTCCCCTCCATCGTG	AGGGTGAGGATGCCTCTCTT
*ACTG1*	TGTTTCCTTCCATCGTCGGG	GCCATGCTCAATGGGGTACT
*CTGF*	TGGTCCTCCTCGCCCTC	GTCCAGCACGAGGCTCAC
*COL1A1*	TTCAGCTTTGTGGACCTCCG	TGGGATGTCTTCGTCTTGGC
*CYR61*	CGCCTTAGTCGTCACCCTTC	CTTGGCGCAGACCTTACAGC
*DUSP1*	GCGCAAGTCTTCTTCCTCAAAG	CCATGGGGGTCGACTGTTT
*FN1*	AAGAGGCAGGCTCAGCAAAT	TCGCAGTTAAAACCTCGGCT
*MYL9*	CACGACATGCTGGCCTCG	GGGCCTCGCTCATCATGC
*P16/INK4*	GCTAGAGAGGATCTTGAGAAGAGG	GGCACGATGTCTTGATGTCCCC
*SerpinE1*	GCAGCAGATTCAAGCAGCTAT	CTCCTTGTACAGATGCCG
*TBP*	TGACCCAGGGTGCCATGA	GGGTCAGTCCAGTGCCATAA
*THBS1*	GCATCACCCTGTTTGTGCAG	GAAGACGCTTTGGATGGGGA
*TPM1*	CAAGAAGGCGGCGGAAGA	TCTTCGGTGCCCTTGAGTTT
*TPM2*	TCTGAATCCGTGAAGGAGGC	CCACATCTGCCTCAGCATCA

Left lane: forward primer, right lane: reverse primer.

#### RNA-seq

RNA was isolated with Trizol from HGPS and age-matched control cells. One microgram of RNA was sent to the Institute of Applied Genomics (Udine, Italy) for deep sequencing. cDNA libraries were processed accordingly with the standard Illumina protocol and sequenced with the HiSeq2500 (4-plex run, 1 × 50 bp reads, about 30 M reads/sample). Reads were aligned to the UCSC hg19 version of the human genome using Tophat2 ([56]; v2.1.1), with parameters -g 1 --segment-length 24 --library-type fr-secondstrand --no-coverage-search, and quantified to the hg19 UCSC genes with HTSeq-count ([57]; v0.5.4p5) with parameters -m union -s yes.

Differential expression analysis was performed in R (v3.5.1) using DESeq2 ([58]; v1.20.0). Counts data from all conditions were filtered based on their raw count, keeping only those where the sum of the counts for all samples was >1. Genes were considered differentially expressed with Benjamini–Hochberg adjusted *p*-value (FDR) < 0.05. Ingenuity Pathway Analysis (IPA [Qiagen, http://www.qiagen.com/ingenuity]) was used to perform gene ontology.

#### Analysis of PA_s_ activities by zymography

Enzymatic activity of PA was assayed according to the method of Shimada and colleagues [59] using a chromogenic substrate (substrate d-val-leulys-*p*-nitroanilide) assay. Samples were incubated with plasminogen, and the absorbance generated at 405 nm, related to PA activity, was normalized to cells present in the culture dish. To characterize the type of PA present in the samples, aliquots of conditioned medium were separated by 10% sodium dodecyl sulfate polyacrylamide gel electrophoresis (SDS-PAGE) under non-reducing conditions. After electrophoresis, gel was washed in 2,5% TritonX-100 for 30 min and then in water for 30 min. PA was then visualized by placing the TritonX-100 washed gel on a casein-agar-plasminogen underlay, as previously described [60]. All the bands were plasminogen dependent. PAI-1 was detected by reverse zymographic assays. 0.05 U/ml human urokinase (Serono, Denens, Switzerland) was added to the casein-agar-plasminogen underlay as previously described [61]. Molecular weights were calculated from the position of pre-stained molecular weight markers subjected to electrophoresis in parallel lanes. Analysis of PAs activities by zymography are representative of at least three different experiments.

#### Beta-galactosidase (beta-Gal) staining

HGPS fibroblasts were fixed in 4% formaldehyde and incubated at 37 °C without CO_2_ with the following solution: 1 mg/ml *X*-*gal* (5-bromo-4-chloro-3-indolyl-b-D-galactopyranoside), 5 mM potassium ferrocyanide, 5 mM potassium ferricyanide, 150 mM NaCl, 2 mM MgCl2 in 40 mM citric acid/sodium phosphate pH 6.0. Staining was evident within 24 h. Beta-Gal staining analysis are representative of at least three different experiments.

#### Morphometric analysis

To examine the overall percentage of blebbed nuclei in cells not treated and treated with TM5441, we performed a morphometric analysis in which nuclei were classified as blebbed if they contained three or more lobulations. Two independent observers performed this classification in a blind manner and the independent datasets were then averaged. To calculate circularity, 137 nuclei for each sample were analized. Circularity was calculated using a built-in function of ImageJ with the formula: 4π(area/perimeter^2). A value from 0 to 1 was assigned to each nucleus, where 1 represents a perfect circle and values closer to zero represent increasingly deformated nuclei.

#### Alkaline comet assay

2YO and 8YO HGPS were untreated or treated with TM5441 for 10 days. Single cells were analyzed for DNA breaks as previously described [62] employing the ‘TriTek Cometscore version 2.0’ software (Tritek Corporation, Sumerduck, VA, USA). The percentage of tail DNA was used as the measure of DNA damage. One hundred cells for each experimental point were scored.

#### Bioenergetic analysis

Mitochondrial function and energy phenotype were determined using a Seahorse XF96e Analyzer (Seahorse Bioscience—Agilent, Santa Clara CA, USA). The fibroblasts were plated at the density of 15 × 10^4^. Mitochondrial stress test was performed according to Agilent’s recommendations. Briefly, growth medium was replaced with XF test medium (Eagle’s modified Dulbecco’s medium, 0 mM glucose, pH = 7.4; Agilent Seahorse) supplemented with 1 mM pyruvate, 10 mM glucose and 2 mM L-glutamine. Before the assay the fibroblasts were incubated in 37 °C incubator without CO_2_ for 1 h to allow to pre-equilibrate with the assay medium. The test was performed by measuring at first the baseline oxygen consumption rate (OCR), followed by sequential OCR measurements after injection of oligomycin (1,5 µM), carbonyl cyanide 4‐ (trifluoromethoxy) phenylhydrazone (1 µM) and Rotenone (0,5 µM) + Antimycin A (0,5 µM). This allowed to obtain the key parameters of the mitochondrial function including basal respiration, ATP-linked respiration, maximal respiration and spare respiratory capacity.

Cell energy phenotype test was performed according to Agilent’s recommendations. Briefly, growth medium was replaced with XF test medium (Eagle’s modified Dulbecco’s medium, 0 mM glucose, pH = 7.4; Agilent Seahorse) supplemented as described above. The Cell Energy Phenotype Test Kit measures the basal OCR and then OCR after the injection of the stressor mix (1 μM of Oligomycin and 1 μM of FCCP). Oligomycin inhibits mitochondrial ATP production causing a compensatory increase in glycolysis rate measured as the Extracellular Acidification Rate (ECAR). FCCP depolarizes the mitochondrial membrane forcing the OCR to the maximum values. OCR/ECAR ratio, determined from the normalized OCR and normalized ECAR, indicating the cellular metabolic potential, were evaluated from the Cell Energy Phenotype Test data. All data were analyzed with XFe Wave software. Bioenergetic analysis are representative of at least three different experiments.

### Statistical analysis and reproducibility

Data are presented as mean ± SD of at least three independent biological replicates. The number (*n*) of independent experimental replications are reported in the figure legends.

Statistical analysis was conducted using Prism 7.0 A software (Pad Software). All data meet the assumptions of the tests (e.g., normal distribution). Statistical significance was determined using unpaired, two-tailed Student’s *t* test to compare the means of two groups, while One-way ANOVA or Two-way ANOVA were used, respectively, for comparison among the different groups and to examines the influence of two different independent variables on dependent variable. Tukey’s test was used for multiple comparison analysis.

Statistical significance was defined as *P* < 0.05 (*), *P* < 0.01 (**), and *P* < 0.001 (***).

## Supplementary information

Supplementary Figure Legends

Supplementary Figure 1

Supplementary Figure 2

Supplementary Figure 3

aj-checklist

Original Data File

## Data Availability

The RNA-seq data supporting the findings of this study are openly available in the SRA database (https://www.ncbi.nlm.nih.gov/sra), with accession number PRJNA526216.
