# Supplementary material for: SerpinE1 drives a cell-autonomous pathogenic signaling in Hutchinson–Gilford progeria syndrome
Source: Cell Death Dis. 2022 Aug 26;13(8):737. doi: 10.1038/s41419-022-05168-y (PMC9418244; doi:10.1038/s41419-022-05168-y)
Supplement: Supplementary file 1 — Supplementary Figure Legends [file 41419_2022_5168_MOESM1_ESM.docx]

**Supplementary figure legends**

**Supplementary Figure 1.**

**DNA damage accumulates precociously in cultured primary human HGPS compared to control fibroblasts.**

**A.** Beta-Gal staining in Ctrl, 2YO and 8YO HGPS fibroblasts. Quantification of the percentage of beta-gal positive cells of Ctrl, 2YO and 8YO HGPS fibroblasts.

**B.** qRT-PCR for *p16/INK4* (n=3) and *p21/Waf1 (n=3)* in 8YO control (Ctrl), 2YO and 8YO HGPS cells.

**C.** Cropped immunoblot for P16, p53 phosphorylated at Serine 15 (Ser15 P53) and Tubulin (TUB) in 8YO control (Ctrl), 2YO and 8YO HGPS. Plots represent P16/TUB ratio and Ser15 P53/TUB ratio based on the average for each experimental point, (n = 3).

**D.** Cropped immunoblot for P21 and GAPDH in 8YO control (Ctrl), 2YO and 8YO HGPS cells. Plot represents P21/GAPDH ratio based on the average for each experimental point, (n = 3).

**E.** Representative images of immunostaining for Phospho-NbsI (P-Nbs1) (green), γ-H2AX (red) and DAPI (blue) in 2YO HGPS and age matched control fibroblasts (2YO Ctrl) at different passage (p13, p14 and p15). Scale bar 50μM. Quantification of the percentage of nuclei with at least three foci positive for P-Nbs1 and γ-H2AX, (n=3).

**F.** Representative images of immunostaining for P-NbsI (green), γ-H2AX (red) and DAPI (blue) in 8YO HGPS and age matched control fibroblasts (8YO Ctrl) at different passage (p13, p14 and p15). Scale bar 50μM. Quantification of the percentage of nuclei with at least three foci positive for P-NbsI and γ-H2AX, (n=3).

**G.** Representative images of immunostaining for 53BP1 (red) and DAPI (blue) in HGPS fibroblasts isolated from 2YO patient (HGPS) and age matched control fibroblasts (Ctrl) (Top panels) and 8YO patient (HGPS) and age matched control fibroblasts (Ctrl) (Bottom panels). Scale bar 30μM. Quantification of the percentage of nuclei with at least three foci positive for 53BP1in 2YO patient (HGPS) and age matched control fibroblasts (Ctrl) (Left graph) and in 8YO patient (HGPS) and age matched control fibroblasts (Ctrl) (Right graph) (n=3).

**Supplementary Figure 2.**

**SerpinE1/PAI-1 expression and activity increase in HGPS primary cells.**

**A.** qRT-PCR for SerpinE1 in control (Ctrl) and fibroblasts isolated from different HGPS patients: HGADFN 188 (2YO), HGADFN127 (3YO), HGADFN367 (3YO), HGADFN169 (8YO), HGADFN167 (8YO) and AG11513 (8YO), (n=3).

**B.** Reverse zymography for SerpinE1 (PAI-1) in 8YO HGPS fibroblasts (HGPS) and age-matched control (Ctrl).

**C.** Zymography for PA/PAI-1 complex and uPA in 8YO HGPS fibroblasts and age-matched control (Ctrl).

**D.** Chromogenic substrate assay in 8YO HGPS fibroblasts (HGPS) and age-matched control (Ctrl).

**E.** Amiloride treatment in control fibroblasts (Ctrl).

**Supplementary Figure 3.**

**TM5441 is effective in restoring SerpinE1/PAI-1 activity in HGPS.**

**A.** Zymography for PA/PAI-1 complex and uPA in 8YO HGPS fibroblasts (HGPS) and age-matched control (Ctrl) untreated (NT) and treated with TM5441 (TM). Quantification of the PA activity, (n=3).
