# Supplementary figures and images for: SerpinE1 drives a cell-autonomous pathogenic signaling in Hutchinson–Gilford progeria syndrome
Source: Cell Death Dis. 2022 Aug 26;13(8):737. doi: 10.1038/s41419-022-05168-y (PMC9418244; doi:10.1038/s41419-022-05168-y)

# Supplementary Figure 1

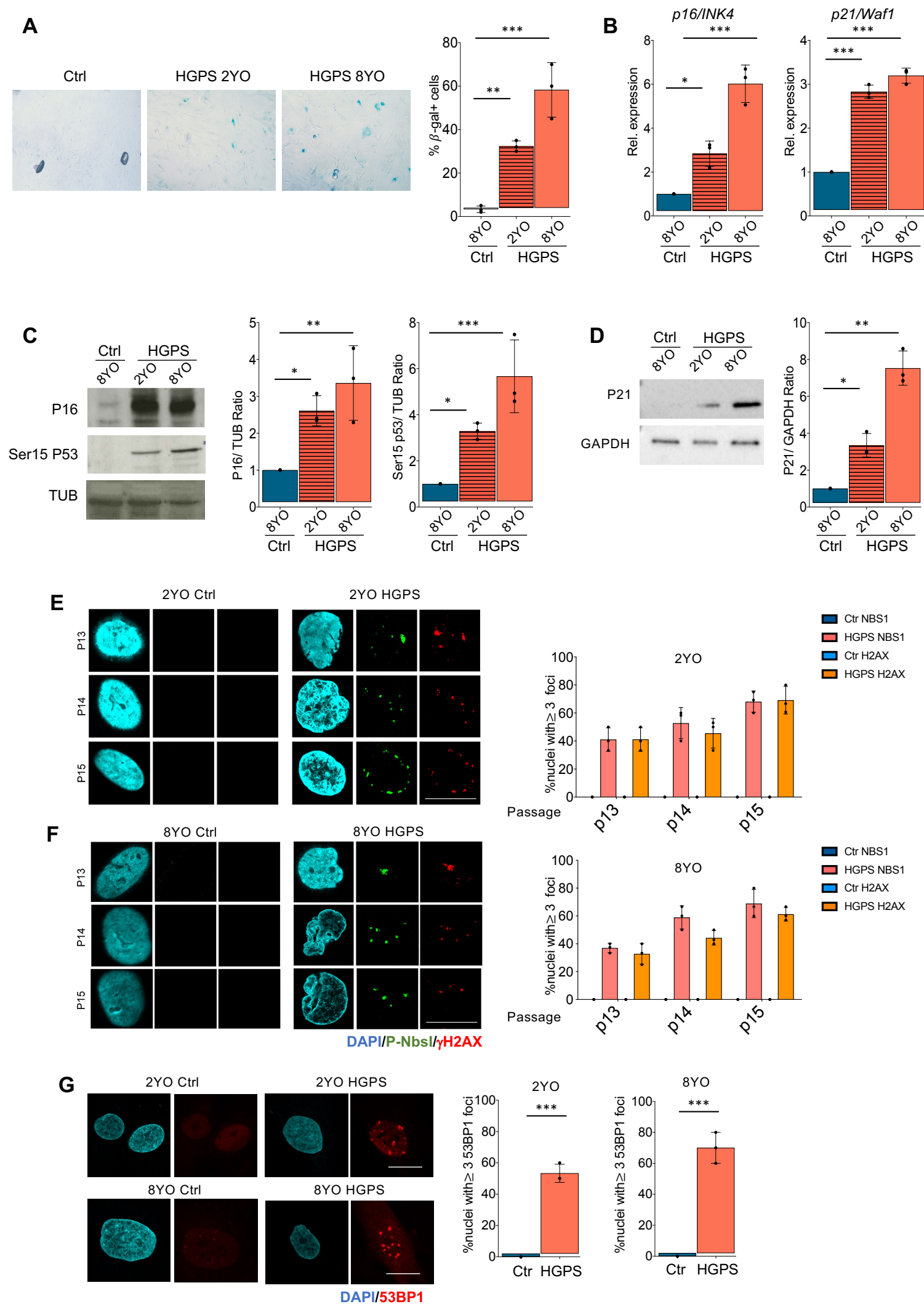

Supplement: Supplementary file 2 — Supplementary Figure 1 [file 41419_2022_5168_MOESM2_ESM.pdf]

Supplementary Figure 2

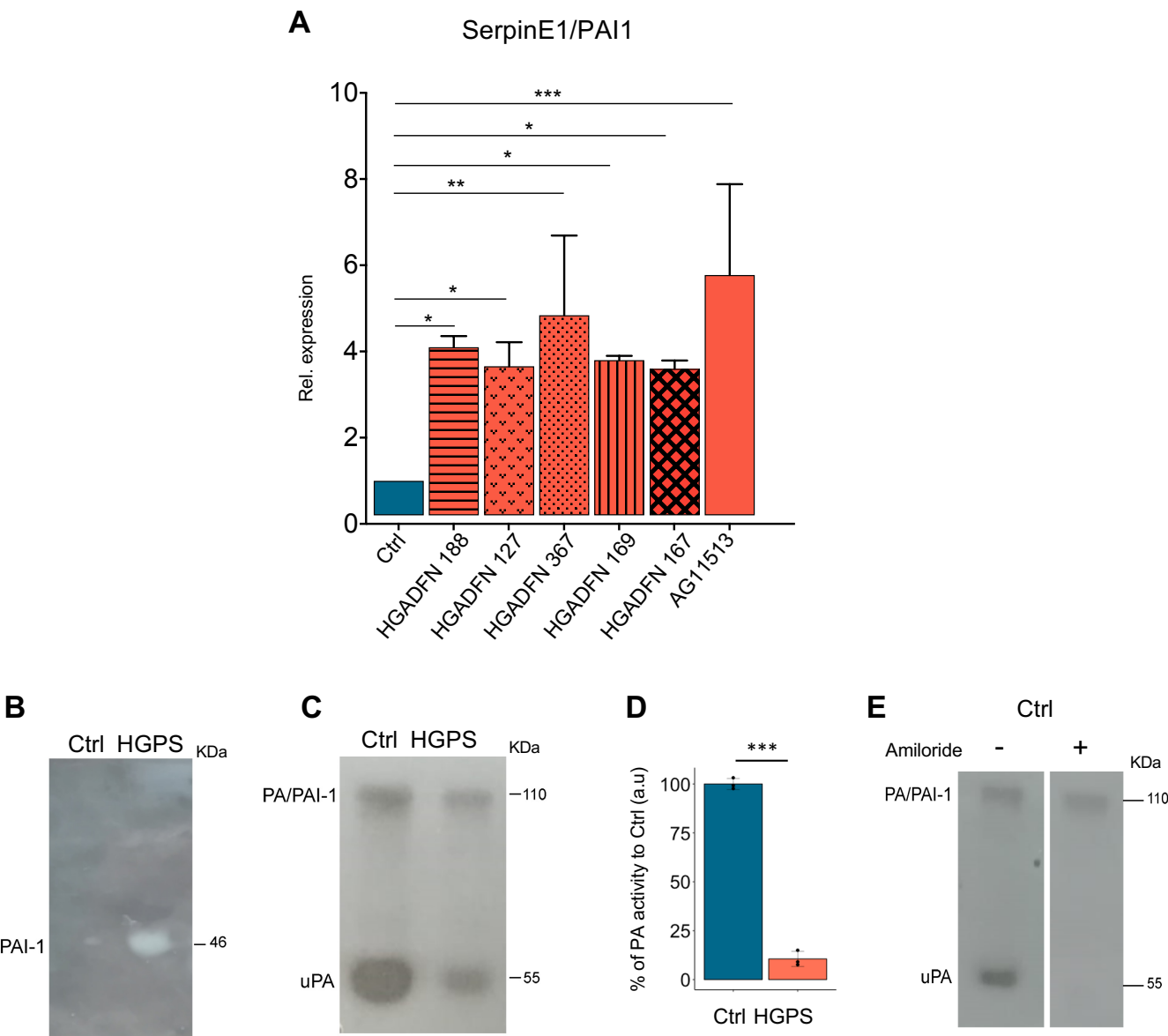

Supplement: Supplementary file 3 — Supplementary Figure 2 [file 41419_2022_5168_MOESM3_ESM.pdf]

Supplementary Figure 3

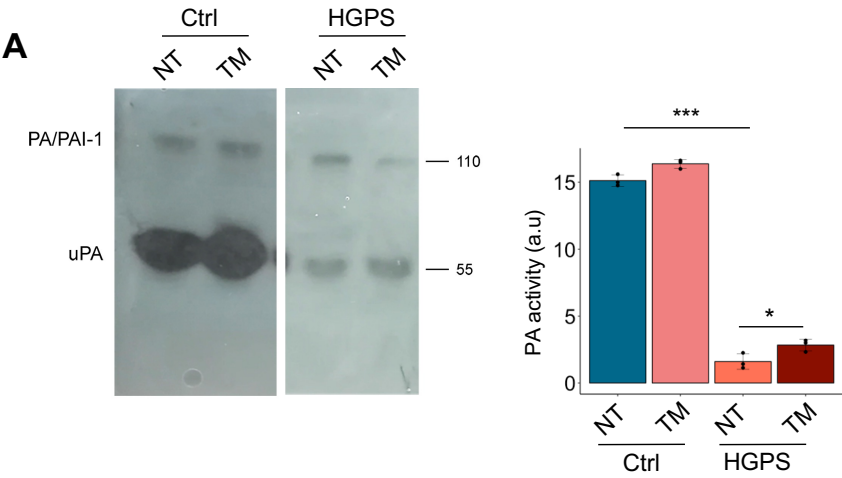

Supplement: Supplementary file 4 — Supplementary Figure 3 [file 41419_2022_5168_MOESM4_ESM.pdf]
