# Supplementary material for: SerpinE1 drives a cell-autonomous pathogenic signaling in Hutchinson–Gilford progeria syndrome
Source: Cell Death Dis. 2022 Aug 26;13(8):737. doi: 10.1038/s41419-022-05168-y (PMC9418244; doi:10.1038/s41419-022-05168-y)
Supplement: Supplementary file 6 — Original Data File [file 41419_2022_5168_MOESM6_ESM.pdf]

Fig.1E

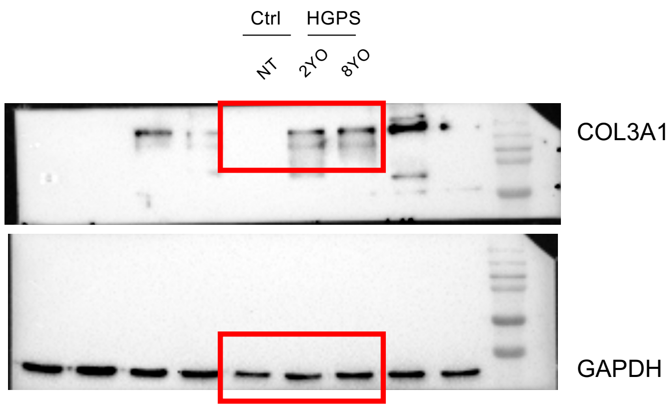

Ctrl
HGPS
NT
2YO
8YO
COL3A1
GAPDH

Fig.1F

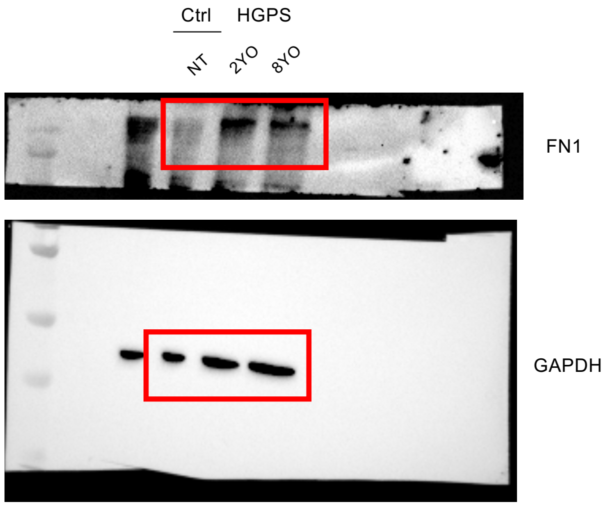

Ctrl
HGPS
NT
2YO
8YO
FN1
GAPDH

Fig.2C

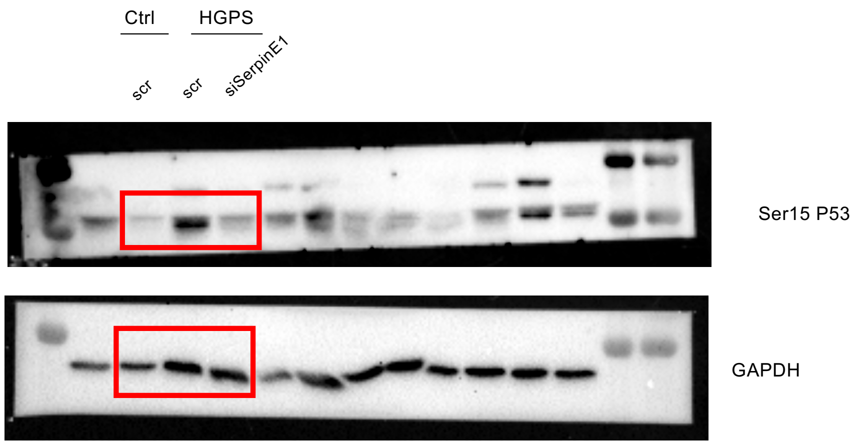
Ctrl
HGPS
scr
scr
siSerpinE1
Ser15 P53
GAPDH

Fig.2G

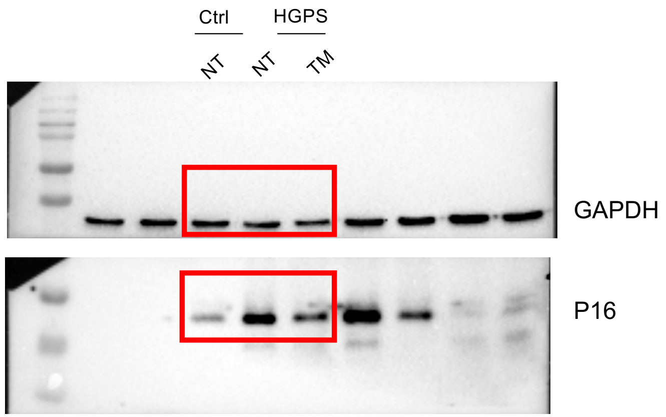
Ctrl
HGPS
NT
NT
TM
GAPDH
P16

Fig.3A

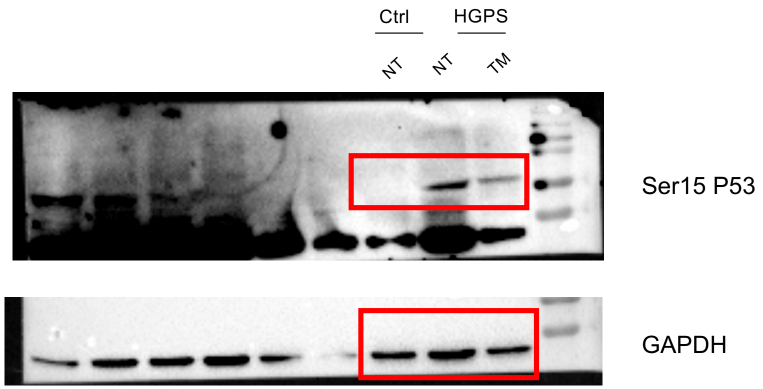

Fig.4E

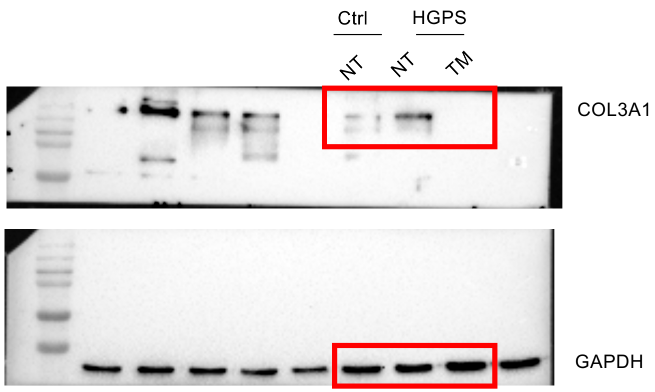

Fig.Supp1C

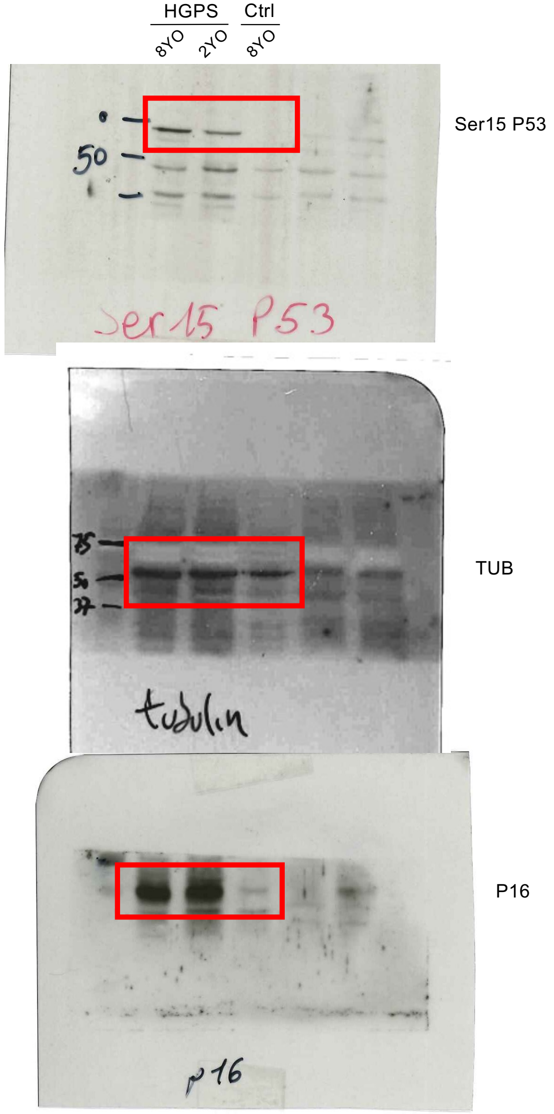

Fig.Supp1D

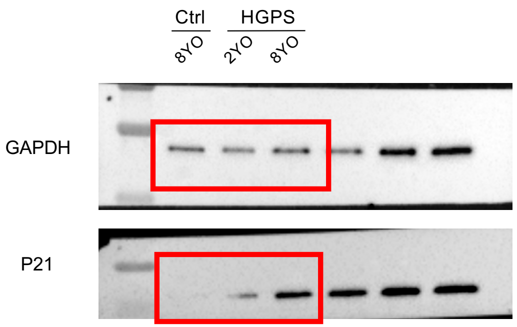

Supp Fig 2B

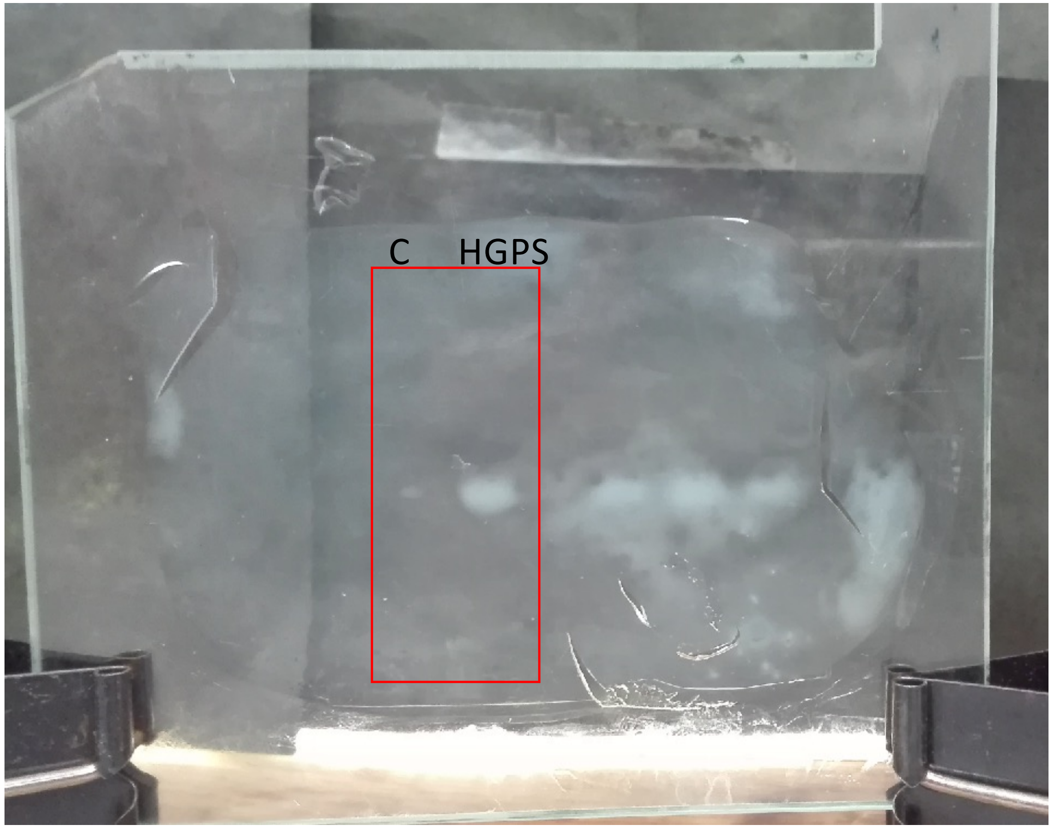

Supp Fig 2C

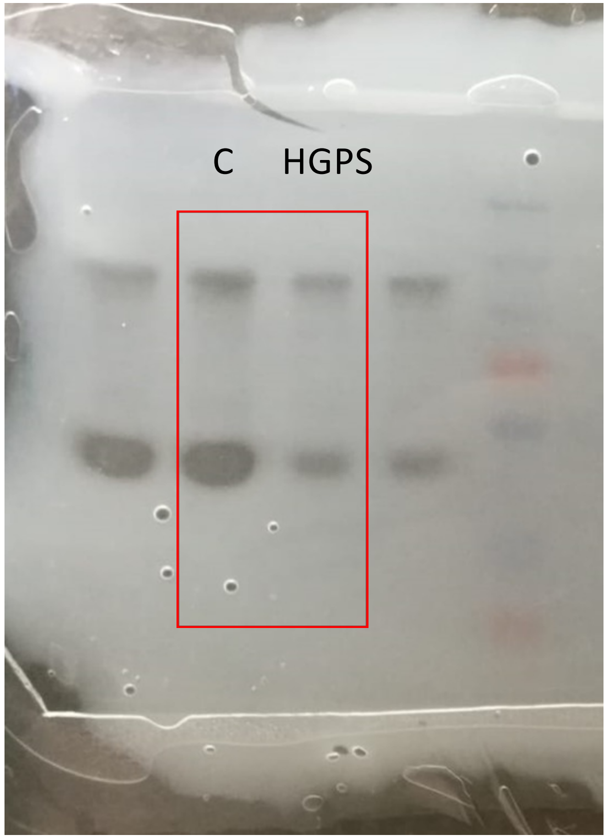

Supp Fig 2E

-Amiloride +Amiloride

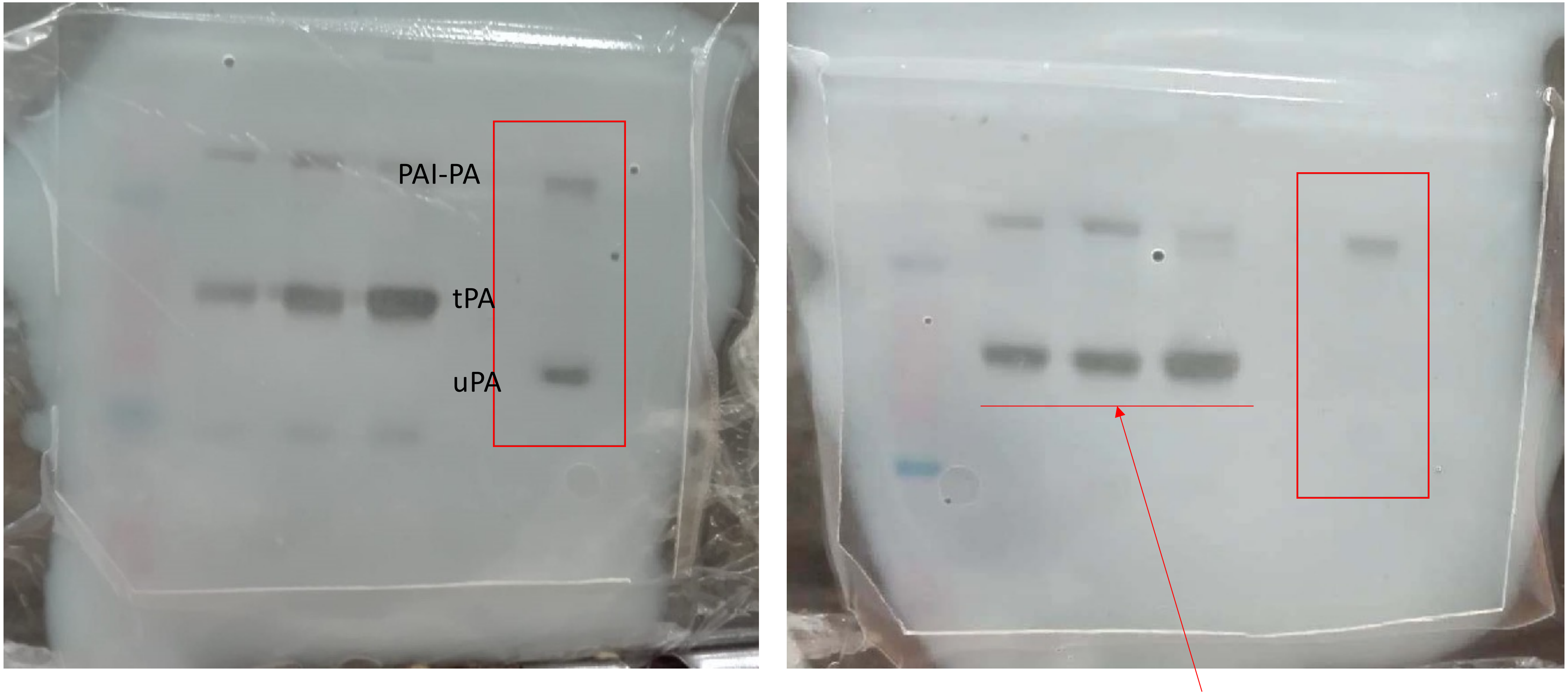

Controls for amiloride
tPA is not affected by the presence of amiloride

Supp Fig 3A

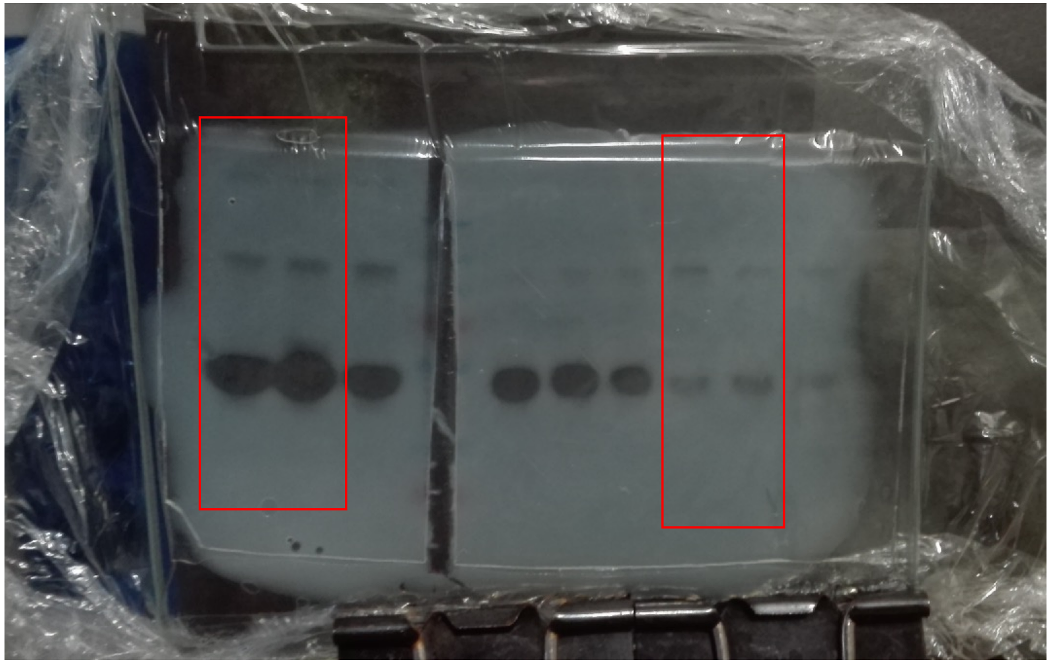

002 2% NT	002 2% TM10	002 2% TM50	002 2% NT	002 2% TM10	002 2% TM50	Ag 2% NT	Ag 2% TM10	Ag 2% TM50
